# Vaccination ecosystem health check: achieving impact today and sustainability for tomorrow

**DOI:** 10.1186/s12919-016-0069-y

**Published:** 2017-01-27

**Authors:** Mitra Saadatian-Elahi, David Bloom, Stanley Plotkin, Valentina Picot, Jacques Louis, Michael Watson

**Affiliations:** 1 Groupement Hospitalier Edouard Herriot, Service d’Hygiène, Epidémiologie et Prévention, Bâtiment 1, 5, place d’Arsonval, 69437 Lyon, Cedex 03 France; 2 Harvard T.H. Chan School of Public Health, 665 Huntington Avenue, Boston, MA 02115 USA; 3 University of Pennsylvania and Vaxconsult, LLC, Philadelphia, USA; 4 Fondation Mérieux, 17 rue Bourgelat, 69002 Lyon, France; 5 Valera, Cambridge, MA 02141 USA

**Keywords:** Conference report, Vaccination ecosystem, Vaccine demand and supply, Vaccine research and development

## Abstract

**Background:**

Vaccination is a complex ecosystem with several components that interact with one another and with the environment. Today’s vaccine ecosystem is defined by the pursuit of polio eradication, the drive to get as many of the new vaccines to as many people as possible and the research and development against immunologically challenging diseases. Despite these successes, vaccine ecosystem is facing keys issues with regard to supply/distribution and cost/profitability asymmetry that risk slowing its global growth.

The conference “Vaccination ecosystem health check: achieving impact today and sustainability for tomorrow” held in Annecy-France (January 19–21, 2015) took stock of the health of today’s vaccination ecosystem and its ability to reliably and sustainably supply high-quality vaccines while investing in tomorrow’s needed innovation.

**Main findings:**

Small and decreasing numbers of suppliers/manufacturing facilities; paucity of research-driven companies; regulatory pressures; market uncertainties; political prioritization; anti-vaccine movements/complacency; and technological and programmatic issues were acknowledged as the major challenges that could weaken today’s vaccination ecosystem. The expert panel discussed also drivers and barriers to a sustainable vaccination ecosystem; the metrics of a vaccination ecosystem; and what should be added, removed, increased, or reduced to maintain the health of the vaccination ecosystem.

**Electronic supplementary material:**

The online version of this article (doi:10.1186/s12919-016-0069-y) contains supplementary material, which is available to authorized users.

## Introduction

Vaccination comprises a complex ecosystem similar to natural ecosystems; it can be represented as an organism-community of international organizations, vaccine manufacturers, donor agencies, foundations, nongovernmental organisms, etc. that interact with one another and with the environment. As a consequence, modifications or variations in one of its components can affect the ecosystem balance.

National immunization programs together with Gavi, the Vaccine Alliance (Gavi), international aid, philanthropic donors, tiered pricing by large research and development (R&D) vaccine manufacturers, and the availability of generic vaccines have significantly decreased vaccine access gaps between high- and low-income countries by improving vaccination of the world’s poorest children. However, this success should not prevent continuous examination of whether we are fully optimizing the huge untapped potential of today’s vaccines without damaging the benefit that future vaccination innovation can bring.

Today’s vaccine ecosystem is defined by the pursuit of polio eradication, the drive to get as many of the new vaccines to as many people as possible and the R&D pursuit of vaccines against immunologically challenging diseases such as HIV, Malaria, TB, Dengue, RSV, HSV, Chlamydia and others. This era is also characterized by the emergence of major private donors and government subsidies. These donors and their agencies aim to extend global access to all vaccines through «push and pull» innovation in vaccines and vaccines systems. To achieve this they implement “market-shaping” aimed at incentivizing increased volumes, lower prices and new producers of high volume, low-cost versions of existing vaccines.

To take stock of the health of today’s vaccination ecosystem and its ability to reliably and sustainably supply high-quality vaccines while investing in tomorrow’s needed innovation, Fondation Mérieux organized a conference entitled “Vaccination ecosystem health check: achieving impact today and sustainability for tomorrow” on January 19–21, 2015, in Annecy, France. Participants included representatives from international organizations [WHO, Gavi, international facility for the purchase of drugs against HIV/AIDS, Malaria and Tuberculosis (UNITAID), Médecins Sans Frontières (MSF), etc.] and industry.

To elicit more consideration of the vaccine ecosystem, a breakout session was held for participants in small working groups to discuss how to keep the vaccination ecosystem healthy.

This report summarizes selected issues discussed by participants, including the key findings and recommendations for future approaches to addressing this issue.

## Vaccination ecosystem

**Fig. 1:**
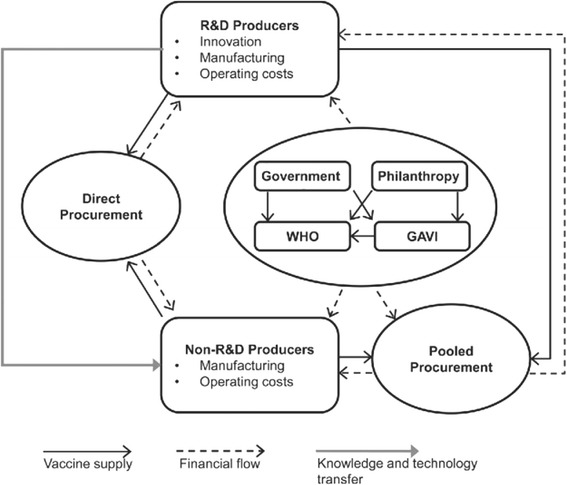
Vaccine ecosystem, a simplified model [1]

The vaccine ecosystem involves a large number of actors (producers, purchasers, payers, supply side facilitators, program implementers, etc.) with mutual interactions (Fig. 1) [1]. Vaccine producers are conventionally divided into two groups: those investing large parts of their revenues in research and development (R&D) and those primarily involved in manufacturing and sales. The first group mainly consists of multinational firms based in the United States and Europe, even if emerging manufacturers from developing countries are scaling up. Governments, philanthropic organizations, WHO, and Gavi ensure vaccine purchasing, while the United Nations Children’s Fund (UNICEF) is the global lead for vaccine procurement.

The Gavi Alliance mainly ensures access to immunization in poor countries. Currently, Gavi supports routine vaccination programs (pentavalent, pneumococcal, yellow fever, etc.), vaccination campaigns (yellow fever, Meningococcal A, measles-rubella, Japanese encephalitis), stockpile for outbreak responses (yellow fever, meningitis, cholera) and malaria vaccine pilot, supply and procurement strategy. UNICEF works in partnership with governments, global partners, and a large number of suppliers. The continuous changes in both supply and demand creates a dynamic market that requires continuous monitoring and management. Indeed, the annual vaccine procurement has increased significantly since 2000 due to the introduction of new vaccines. In 2014, UNICEF procured 2.70 billion doses of more than 40 vaccines for 100 countries at a value of $1.474 billion. Currently, the largest contributions come from industrialized countries, but emerging markets’ participation is increasing over time.

The participants attempted to analyze the health of vaccine eco-system in terms of coverage, demand, supply, and R&D.

### Global vaccine coverage, demand, and supply

The establishment of the World Health Organization Expanded Program on Immunization (WHO-EPI) more than 40 years ago, adequate program design and implementation and international cooperation have led not only to increased vaccine coverage rates around the world, but also to reduced disease burden [2–4], and to smaller access inequities between low- and high-income countries even for new vaccines such as hepatitis B and pneumococcal vaccines. Despite these successes, large coverage gaps remain, which will need significant financial resources and political motivations to address. Indeed, the limited number of manufacturers and buyers in parallel to an increasing demand in terms of new vaccines introduced in the WHO-EPI especially in lower-income countries, could create a disconnect between demand and supply.

An important unaddressed challenge is to vaccinate the 20% of the world’s children born each year that do not receive even the most basic vaccinations. In 2011, the Global Vaccine Action Plan (GVAP) for the Decade of Vaccines (DoV) was created with the ambitions to close the equity gap in vaccine delivery and to unleash vaccines’ vast future potential. The new global vaccines and immunization framework provides space for all stakeholders to improve delivery, demand, and research. GVAP mid-point targets were 1) Diphtheria-Pertussis-Tetanus (DTP3) coverage >90% in all countries and >80% in every district by 2015, 2) polio transmission stopped by 2014, 3) maternal and neonatal tetanus eliminated by 2015, 4) measles eliminated in four regions by the end of 2015, 5) rubella eliminated in two regions by the end of 2015, and 6) introduction of one or more underutilized vaccines in low- or middle-income countries by 2015. An independent assessment performed by the Strategic Advisory Group of Experts on Immunization (SAGE) in 2014 expressed concerns about low implementation of GVAP as all targets except the last one were off track.

### Global vaccine research and development

The vaccine industry is entering a period of uncertainty that threatens the new vaccine pipeline. Additional needs for basic science research; continuously rising manufacturing standards and regulatory requirements; the scientific challenge of developing vaccines for unmet needs (cytomegalovirus, HIV, tuberculosis, etc.); and increased cost, time, and risk of vaccine research and development driven by late-stage (phase 3) trials are among major contributing factors that limit the number of vaccines that a company is willing or able to develop to meet public health needs. One important result of this situation is that major companies are only interested in blockbusters. Therefore, main vaccine manufacturers do not consider many infections with somewhat restricted market size such as Lyme, cryptosporidium, and West Nile a top priority. Furthermore, the pressure to keep vaccine prices low, both in developed and developing countries, threatens to move manufacturing capability out of North America and Europe to reduce costs.

### Today’s vaccination ecosystem health status

Today’s vaccine ecosystem is facing keys issues with regard to supply/distribution and cost/profitability asymmetry that risk slowing the global health of vaccine ecosystem. Indeed, a disconnect exists between the WHO global vaccine action plan (GVAP) and market shaping This could be due to overdependence on “price reduction” metrics and underdeveloped metrics for future innovation and supply security. Furthermore, cost/profitability asymmetries could provoke market exit and market failure when reinvestment is required. Several years of yellow fever vaccine shortage illustrates this situation well [5, 6]. New challenges related to population growth; increase in the number, doses, and cost of vaccines targeting a wider age group beyond that of infants; inadequate systems (trained human resources, supply chain, information system); vaccine supply and price (i.e., availability, financing, pricing, and procurement); and the goal of reaching the 20% of the world’s children born each year that are never (or incompletely) vaccinated due to political crisis, migration, failure of integration (i.e., missed opportunities), hesitancy, and refusal, further amplify the complexity of the vaccination ecosystem.

## Possible solution frameworks

Possible solutions discussed during the conference for sustaining a healthy vaccination ecosystem are summarized in the following paragraphs.

### Global vaccine coverage, demand, and supply

High-quality data are crucial to empower immunization program management, to increase vaccine uptake and to reach those children who are never vaccinated. Regular data review, data triangulation with other available sources, periodic data quality assessment, and training of health care workers are examples of methods that could considerably improve data quality. Household surveys to measure immunization coverage are also needed to validate administrative data and provide additional information on missed opportunities and timely vaccination. Using traditional methods such as home-based vaccination records and new technologies such as modern Information, Communication and Technology (ICT) tools can also considerably improve data quality and thus contribute to improvement in coverage.

Another issue that can influence global vaccine coverage concerns the sustainability of financial support for Gavi–eligible graduating countries. Price-freezing for a period of five years is a possible solution, currently under evaluation, for these countries to sustain their expanded immunization programs without facing huge economic impact.

Weak immunization supply chain systems are another bottleneck to achieving immunization goals. Fortunately, key supply chain challenges have been diagnosed and their importance acknowledged. New technologies to reduce storage volume, expand the reach of cold chain to areas without reliable energy sources using solar equipment or long-lasting vaccine containers, provide new vaccine delivery models, allow combined vaccines and better schedules (number or timing of doses) are some technical solutions that exist and/or are in development and that could address vaccine supply challenges.

As described previously, the existing disconnects between demand and supplies are due to the limited number of manufacturers and buyers and the increasing demand in terms of vaccine products. Gavi adopted a supply and procurement strategic goal to shape the vaccine market by balancing supply and demand, ensuring security of supply, minimizing the cost of vaccines, and fostering development of appropriate and quality vaccines. So far, Gavi increased the number of vaccine suppliers from five in 2001 to 15 in 2014, including manufacturers from emerging markets. Other procurement strategies could further improve vaccine supply, including negotiation such as selective contracting/exclusive dealing and auctions.

### Global vaccine research and development

Possible solutions for sustaining vaccine research and development and innovation are 1) incentives for industry to invest in new vaccines and improve old vaccines, 2) agreement between Food and drug administration/European medicine agency (FDA/EMA) to limit numbers needed for efficacy trials (phase 2B versus phase 3 trials), 3) increased interaction between high- and low-income countries to facilitate technology transfer, and 4) set-up of an international vaccine development fund that would support biotech escalating from lab to proof of concept and vaccine manufacturers developing vaccines for tropical and neglected diseases. Policies that make innovation and distribution reasonably profitable could also address these issues. Such policies include providing government supports for development and production, reducing uncertainty by establishing clear property rights, taxing negative spillovers, and subsidizing positive spillovers. Innovation must be profitable, but regulation to lower prices can slow innovation and diffusion by reducing return on investment. A price that is affordable to governments and donors and reasonably covers manufacturer’s minimum requirement could thus enhance vaccine R&D. Price discrimination, i.e., different prices for the same product in different settings (e.g., developed vs. developing countries), could also allow profitability to be maintained, thus generating more innovation. In low-income countries, differential patent rights, compulsory licenses, prizes, and advance market commitments to recommend a vaccine once licensed could also address supply and innovation.

Conditional licensure of vaccines as part of a system that would incorporate a requirement for rapid cycle and other real-time evaluations following introduction is another potential solution. Indeed, the cost and time of large routine safety trials prior to vaccine licensure are constantly increasing and could have a negative impact on the vaccine development pipeline without concomitant and dramatic increase in our ability to assess safety. The examples of Rotateq and Rotarix show that despite very large phase 3 clinical trials, increased risk of intussusception only became apparent and statistically significant in the post licensure period (Table 1).

**Table 1:** Risk of intussusception following rotavirus vaccination

Vaccine	Phase III Clinical Trial	Post introduction
Rotateq	RR = 1.6 (95% CI: 0.4–6.4) [10]	RR = 9.9 (95% CI: 3.7–26.4) [11]
Rotarix	RR = 0.86 (p = ns) [12]	RR = 6.8 (95% CI:2.4–19.0) [11]

*RR* Relative risk, *CI* Confidence interval, *ns* not statistically significant

Similarly, post-introduction data on pneumococcal conjugated vaccines (PCV) show that the vaccine had higher impact on the reduction of invasive pneumococcal diseases, otitis media, and all causes of pneumonia than what was reported in phase III clinical trials (Table 2).

**Table 2:** Comparison of phase III clinical trials and post marketing data on PCV efficacy

Outcome	Phase III Clinical Trial	Post introduction
IPD	93.9% (95% CI: 79.6–98.5) [13]	>99% [14]
Otitis media	7.0% (95% CI: 4.1–9.7) [13]	20% (95% CI: 4–34) [15]
All cause pneumonia (<5Y)	6% (95% CI:-1.5–11) [16]	39% (95% CI: 22–52) [17]
Cost-effectiveness	US$80,000/QALY [18]	US$75,000/QALY [19]

*IPD* Invasive pneumococcal diseases, *CI* Confidence interval, *QALY* Quality-adjusted life year

Conditional licensure will lower the financial barriers to development of new vaccines and consequently allow a broader portfolio of vaccines to be developed.

### Valuing vaccination

In recent years, a compelling line of inquiry has established the economic benefits of health, at both individual and aggregate levels. Most economic evaluations of vaccines focus on a narrow set of vaccination-mediated benefits (i.e., avoided medical costs and absenteeism) and fail to account for the role of vaccination as a driver of wealth and its known impact on health, leading to potentially sizable undervaluation of the true economic value of vaccines. A broader perspective in economic analysis considers other benefit categories including 1) outcome-related productivity gains, 2) behavior-related productivity gains, 3) community externalities (i.e., herd effect and prevention of antibiotic resistance), and 4) utilitarian value of health gains. Cost-effectiveness and benefit-cost analyses are tools to assess the value of vaccination. The latter is more appropriate for economic analysis because it looks at a range of health and non-health related outcomes that are expressed in terms of a dollar value. The example of Hib vaccine in children provided evidence of a benefit-cost ratio above 1 [7]. Another example is the case of dengue infection. By taking into account reduced spending on outbreak control, averted losses in tourism flows, and avoided productivity losses due to long-term dengue sequel, introducing dengue vaccine in national immunization programs in Brazil would more than double the narrow benefit calculation, allowing twofold return on the investment [8]. Similar conclusions can be drawn for human papilloma virus (HPV) vaccination if the full benefits of vaccination are captured [9].

Conceptualizing the full benefits of vaccination allows better recognition of the value of vaccination as a driver of both wealth and health. Monetizing the impact of vaccination provides policymakers with sufficient information to make well-informed decisions about including other new vaccines in national immunization programs.

## Outcomes of the workshops

Breakout sessions following the overall presentations on the current status of today’s vaccination ecosystem allowed participants to discuss main topics that deserved more in-depth attention.

Different factors were identified as drivers or barriers that could influence the sustainability of the vaccine ecosystem in terms of vaccine coverage, supply and demand as well as R&D (Additional file 1). The working group identified also several items to improve or add to ensure the health of the vaccination ecosystem (Additional file 2). Furthermore, reduction of the precautionary principle in government regulation and market failure could also contribute to the health of the vaccination ecosystem.

## Conclusions and recommendations

The panel concluded that despite the considerable progress achieved, introduction of new vaccines in LMICs, sustained coverage with new and traditional vaccines, and better performance of supply chains), today’s vaccination ecosystem faces several challenges that could weaken it. These include small and decreasing numbers of suppliers/manufacturing facilities; paucity of research-driven companies; regulatory pressures; market uncertainties (cost-effectiveness, price, higher demand and supply); political prioritization (competing priorities or distortion due to crisis, political instability); anti-vaccine movements/complacency; and technological and programmatic issues (supply chain, cold chain custody, health care worker education).

However, the panel identified several potential solutions. Guaranteed return on investment for donors, governments, and global stakeholders; presence of multiple effective suppliers to reduce monopolies and secure supply; financing for research and development; political prioritization by raising awareness about needs; empowerment of developing country manufacturers (e.g., technology transfer); and balance of public-private perspectives for global public goods and distinction of the roles of each sector could positively affect the vaccination ecosystem.

In parallel, the current low-price-focused market shaping could lead to critical asymmetry between cost and profitability for Gavi–dedicated vaccines. This phenomenon is apparent in the supply shortage for most Gavi vaccines, exit of R&D producers from production to heritage vaccines, increased concentration of most vaccine production by fewer producers, increased number of vaccines with just one or two suppliers, and static or falling levels of vaccine producers and R&D activity. Challenging vaccine prices is complex. The need exists to collect more accurate data to assess the full benefits of vaccines because we cannot challenge the cost without considering the whole benefits. Piggyback analysis, i.e., economic evaluation embedded in clinical trials, would be a good way to quantify economic values of vaccines in terms of productivity, for example. However, this would need expensive long-term follow-up.

To summarize, the concept of an ecosystem is a useful way of thinking about vaccines. A healthy ecosystem should be able to maintain its organization and function over time in the face of external stress. The supply issue is a central aspect that requires much more work. Research and development also face challenges. Prioritizing a list of vaccine-preventable diseases to target and matching it with R&D activities could optimize the latter, especially if a global development fund is in place. Mechanisms such as regulatory reforms and technical innovation to improve access to markets and shorten disruption periods could also contribute to the sustainability of the vaccination ecosystem. Our goal should be to investigate how we can best work together to ensure the sustainability of increasing global vaccination without damaging global vaccine production and/or R&D. A shared responsibility is needed to keep the vaccination ecosystem healthy.

## Additional files

Additional file 1: Drivers and barriers to a sustainable vaccination ecosystem. (DOCX 17 kb)
Additional file 2: Requirements for a healthier vaccination ecosystem. (DOCX 14 kb)
