# Supplementary material for: Vaccination ecosystem health check: achieving impact today and sustainability for tomorrow
Source: BMC Proc. 2017 Jan 27;11(Suppl 2):1. doi: 10.1186/s12919-016-0069-y (PMC5290488; doi:10.1186/s12919-016-0069-y)
Supplement: Additional file 1: — Drivers and barriers to a sustainable vaccination ecosystem. (DOCX 17 kb) [file 12919_2016_69_MOESM1_ESM.docx]

**Additional file 1**: Drivers and barriers to a sustainable vaccination ecosystem

| **DRIVERS** | |
| --- | --- |
| **vaccine coverage, demand and supply** | **Vaccine R&D** |
| Educating health care workers on the value of vaccines | Profit and ROI   - “Efficient” profit—to which suppliers and consumers can agree - ROI for donors and governments and for global stakeholders (e.g., clear public health impact)   Clear business case for individual vaccines, including data on disease burden and cost implications to inform decision making |
| Multiple effective suppliers to reduce monopolies and secure supply | Financing for R&D and the fruits of R&D (recognizing the need for differing options):   - Push investment for R&D (*PDP model for example) - Pull investment for R&D   Incentives that specifically target adapted vaccines for LMICs |
| Adapted procurement policy linked to healthy markets and competition | Political will across the vaccine lifecycle (to invest, implement, evaluate, improve) |
| Prioritization and articulation of public health targets (to inform market opportunities) | Empowering developing country manufacturers (e.g., technology transfer) |
| Balance of public-private perspectives for global public goods and distinction of the roles of each sector |  |
| **BARRIERS** | |
| **vaccine coverage, demand and supply** | **Vaccine R&D** |
| Political prioritization   - Competing priorities or distortion due to crises - Political instability | Market uncertainty   - Epidemiology, cost-effectiveness, demand, price |
| Lack of ability to pay | Regulatory barriers   - Associated costs for compliance   Coherence and convergence of requirements |
| Opaque costs from manufacturers | Technology and disease science |
| Programmatic challenges   - Supply chain and cold chain storage - Health care worker education | Opportunity cost vs. investment in drug development, which can be more lucrative |
| Anti-vaccine movement/complacency |  |

ROI: Return on investments; PDP: Product development partnership; LMICs: Low and middle income countries

*PDPs are private-public partnerships focused on discovery and development of new health technologies/products in particular for developing countries and work as virtual non-profit R&D organizations.
