# Supplementary material for: Vaccination ecosystem health check: achieving impact today and sustainability for tomorrow
Source: BMC Proc. 2017 Jan 27;11(Suppl 2):1. doi: 10.1186/s12919-016-0069-y (PMC5290488; doi:10.1186/s12919-016-0069-y)
Supplement: Additional file 2: — Requirements for a healthier vaccination ecosystem. (DOCX 14 kb) [file 12919_2016_69_MOESM2_ESM.docx]

**Additional file 2**: Requirements for a healthier vaccination ecosystem

| **Factors to improve** | **Factors to add** |
| --- | --- |
| - Research on adjuvants  - R&D on immunologically challenging diseases and unmet medical needs (Ebola, Lyme, chikungunya)  - Improvement of old vaccines (flu, pertussis, BCG)  - Greater price/demand forecast transparency  - More social responsibility  - Technological innovation (e.g., greater thermostability, edible vaccines, new administration devices)  - Flexible contracts/auctions for buyers  - Decoupling R&D from price (with help of push funding and/or other incentives)  - Assessing procurement/distribution costs on a system-wide basis  - Mechanisms to shorten access to markets and disruption periods (regulatory reform, more recognition/reciprocity of WHO prequalification)  - Regulation, e.g., clinical trial design, surrogate endpoints  - ROI (higher returns but lower prices)  - Academia-industry-foundation relationships  - Greater alignment among government entities responsible for vaccine approval (FDA), recommendation (CDC), and reimbursement  - Public understanding  - Philanthropy | - Incentives for more R&D  - Prizes for innovations (but with access provisions), including a more transparent paradigm  - Prioritized list of pathogens to be targeted for specific markets  - Technical advances (e.g., universal flu vaccine)  - Global vaccine development fund for vaccines not valued by manufacturers but needed for serious diseases  - Target product profile and advanced recommendation commitment between organisms such as ACIP, NICE, etc.  - Regulatory reform (e.g., conditional licensure)  - Ways to measure the health of markets and of innovation and how to fix market failure |

FDA: the food and drug administration; CDC: centre for disease control; ACIP: advisory committee on immunization practices; NICE: the national institute for health and care excellence
